# Quantitative lung ultrasound to guide surfactant retreatment in preterm neonates born at ≤30 weeks’ gestation: a multicentre retrospective non-inferiority diagnostic accuracy study

**DOI:** 10.1016/j.ebiom.2025.105865

**Published:** 2025-07-25

**Authors:** Daniele De Luca, Almudena Alonso-Ojembarrena, Davide Sarcina, Irene Gutierrez-Rosa, Barbara Loi, Fiorella Migliaro, Letizia Capasso, Francesco Raimondi

**Affiliations:** a Division of Paediatrics and Neonatal Critical Care, “A. Béclère” Hospital, APHP-Paris Saclay University, Paris, France; b Physiopathology and Therapeutic Innovation Unit-INSERM U999, Paris Saclay University, Paris, France; c Neonatal Intensive Care Unit, Puerta del Mar University Hospital, Cádiz, Spain; d Biomedical Research and Innovation Institute of Cádiz (INiBICA) Research Unit, Puerta del Mar University Hospital, Cádiz, Spain; e Division of Neonatology, Department of Translational Medical Sciences, University of Naples Federico II, Naples, Italy

**Keywords:** Newborn infant, Prematurity, Respiratory distress syndrome, Poractant, Dose, Redosing

## Abstract

**Background:**

The most critically ill neonates may require repeated surfactant doses, but there are no consensus criteria for retreatment. We aim to verify whether lung ultrasound (LUS) aeration score predicts surfactant retreatment with accuracy equal to that known for the first administration.

**Methods:**

This was a multicentre, retrospective, non-inferiority, diagnostic accuracy study. Preterm (≤30 weeks’ gestation) neonates were enrolled and LUS aeration score was calculated at T1 (i.e., ≈1 h of life) and before surfactant administration, if any; and T2: ≈12 h of life and at least 10 h after the first surfactant administration. The area under the curve (AUC) was chosen considering the score as triage (i.e., highest sensitivity) and replacement test (i.e., highest global accuracy).

**Findings:**

AUC was higher for T2 (AUC = 0.854 (95% CI: 0.84; 0.87), *p* < 0.0001) than for T1 score (AUC = 0.69 (95% CI: 0.67; 0.71), *p* < 0.0001; difference = 0.165 (95% CI: 0.116; 0.214), *p* < 0.001). AUC of T2 score was similar to that previously reported at T1 for prediction of the first surfactant treatment in preterm (*p* = 0.225) or in late preterm/term patients (*p* = 0.579). Cut-offs to use the T2 score as triage (score = 4) or replacement (score = 8) test had a sensitivity of 98% and a global accuracy of 78%, respectively. Accuracy is independent of gestational age, and the T2 score is associated with the surfactant retreatment (aOR = 1.57 (95% CI: 1.38; 1.79), *p* < 0.001).

**Interpretation:**

In preterm neonates, LUS aeration score calculated at ≈12 h of life and at least 10 h after the first surfactant administration predicts retreatment with accuracy equal to that of the score calculated at ≈1 h of life to predict the first administration. The accuracy is independent of gestational age.

**Funding:**

None.

**Research in context**

**Evidence before this study**

LUS aeration score is known to be accurate to guide surfactant replacement in neonates, irrespective of gestational age. Little is known about the need for surfactant retreatment. This is relevant because the most immature and sick neonates may require repeated doses, and the retreatment is associated with better clinical response, but it is unclear which patients should be retreated.

**Added value of this study**

This multicentre diagnostic study of 705 preterm neonates (≤30 weeks’ gestation) with respiratory failure early after birth demonstrated that the accuracy of LUS aeration score (calculated at ≈12 h of life and at least 10 h after the first surfactant administration) in guiding the surfactant retreatment was similar to that of the score calculated at ≈1 h of life to guide the first surfactant administration. A score higher than 8, and 4 or lower had the highest global accuracy (replacement test) and highest sensitivity (triage test), respectively. These data were warranted to understand how to personalise surfactant retreatment and expand our knowledge about the ultrasound-guided surfactant replacement.

**Implications of all the available evidence**

The findings are clinically relevant as they demonstrate that LUS aeration score, calculated after the first surfactant administration, can accurately guide surfactant retreatment in preterm neonates.

## Introduction

Point-of-care quantitative lung ultrasound (LUS) allows, through bedside calculation of scores, the objective evaluation of lung aeration and ultimately more refined respiratory care.^1^ Originally described in adult critical care,^2^ the technique, due to its simplicity and reliability, is also currently spreading in neonatology: the first typical neonatal application was the prediction of surfactant replacement. Numerous studies and their meta-analyses have demonstrated the accuracy of quantitative LUS-guided surfactant administration,^3^^,^^4^ and the improvement of the timeliness of surfactant replacement,^5^^,^^6^ without increasing associated costs.^7^ Earlier surfactant administration, when needed, constitutes part of optimal perinatal care to improve major clinical outcomes.^8^

Nevertheless, studies have mainly focused on prediction of the first surfactant dose, and little is known about the need for surfactant redosing. This is relevant because the most immature and sickest neonates may require repeated doses as their lung inflammation may inactivate exogenous surfactant,^9^ decrease lung aeration and eventually cause alveolar collapse. Repeated surfactant treatment is associated with better clinical response,^10^ but it is unclear which patients should be retreated. Retreatment is usually decided on the basis of inspired oxygen fraction (FiO_2_) thresholds. Nonetheless, there is no consensus on the threshold to use. Oxygen dependency can also be due to pathophysiological mechanisms unrelated to surfactant dysfunction and the consequently reduced lung aeration. Thus, a bedside point-of-care technique evaluating lung aeration would be useful to clarify patient pathophysiology and the need for surfactant retreatment.

Investigating whether LUS aeration score can identify neonates needing retreatment is not an easy task. In fact, with complete prenatal steroid prophylaxis, optimal perinatal care and respiratory support, most patients only need one dose.^11^ Furthermore, several factors, such as respiratory support policy as well as dose and type of surfactant, might influence LUS findings. These factors make it relatively difficult to conduct a study of sufficient power and quality, hence the current knowledge gap about LUS and surfactant retreatment. We have sought to fill this gap, by designing a multicentre study overcoming some of these issues. We investigated the diagnostic accuracy of LUS aeration score (calculated at two timepoints within the first 24 h of life) in predicting surfactant retreatment in preterm neonates. Our hypothesis was that the accuracy of at least one of the two scores is non-inferior to that already described in the literature to predict the first surfactant dosing.

## Methods

### Study design

This was an international, multicentre, non-inferiority, diagnostic accuracy study conducted in three academic referral neonatal intensive care units (NICUs) in France, Italy and Spain. The study was retrospective since the data were collected after the index test and the reference standard: we took data from electronic patient files of all eligible neonates admitted between January 2021 and December 2024. This period was chosen because there were no changes in routine respiratory care.

### Ethics

The study was observational and pragmatic because, in all recruiting centres, LUS is the first-line imaging technique routinely performed on all NICU-admitted neonates. Thus, we used only data obtained during routine clinical care which was not changed for study purposes. Ethical approval was granted by institutional review boards of the “Puerta del Mar” Hospital, Paris Saclay and Federico II Universities (i.e., each participating centre, reference n. 5495/AO/22, Paris-Saclay-2023-003, NEO-LUS-23-03), and parents were informed of the anonymous use of data, as required by local regulations. Relevant privacy regulations were respected. Manuscript preparation followed STARD guidelines.^12^ The study was not supported by any funder.

### Participants

All neonates consecutively admitted to the recruiting NICUs during the study period were considered eligible if they were at ≤ 30 weeks’ gestation. This gestational age range was chosen because the need for surfactant retreatment is higher at lower gestational age.^13^ Exclusion criteria were major congenital malformations or chromosomal abnormalities, airleaks (i.e., pneumothorax, pneumomediastinum) preventing a comprehensive ultrasound visualization of the lung, need for surgery, pulmonary hypoplasia, pulmonary hypertension (defined as need for nitric oxide).

Respiratory support was shared by all recruiting centres and essentially based on current evidence and international guidelines.^14^^,^^15^ In detail, the initial respiratory support usually consisted of nasal mask-delivered continuous positive airway pressure (CPAP, 6 cmH_2_O) and the first surfactant dose was administered with a customised INSURE procedure.^16^

However, invasive ventilation was used for neonates needing intubation for delivery room resuscitation,^17^ or for patients with severe ongoing respiratory failure unresponsive to the first surfactant dose, including those needing retreatment, as per local practice. Supplemental oxygen was added when the respiratory support in room air was insufficient to achieve preductal haemoglobin saturation (SpO_2_) ≥ 90%. Only porcine surfactant was used (poractant-alfa, Chiesi Farmaceutici®, Parma, Italy); the first and second doses were 200 and 100 mg/kg, respectively. The first surfactant treatment was generally administered upon NICU admission and within the first 3 h of life when LUS aeration score was >8.^11^ In a few severe cases surfactant was administered within the delivery room resuscitation, as recommended by European guidelines.^14^ The retreatment was performed if FiO_2_ was persistently >0.30 to achieve SpO_2_ ≥ 90% and in any case at least 10 h after the first dose and within the first 72 h of life (see below for reference standard details). This was chosen as 10 h represents the median half-life of desaturated phosphatidylcholine in preterm neonates needing multiple surfactant dosing.^18^ No more than two surfactant doses were given. Perinatal care was provided according to current guidelines and patient requirements. Gestational age was considered based on the best obstetric estimate.

### Index test and reference standard

LUS aeration score was the index test to predict surfactant retreatment. We considered it twice, i.e., two quantitative LUS scans were performed at different timepoints. The aeration score was computed on 6 chest zones (3 per side [upper and lower anterior and lateral]) assigning to each area a value of 0–3 (total score ranging from 0 to 18; the higher the score, the worse the lung aeration), based on classic ultrasound semiology (0 for normal, 1 for interstitial-alveolar, 2 for severe interstitial-alveolar (i.e., white lung) pattern and 3 for consolidated areas).^19^ The score was calculated by investigators proficient in the technique (i.e., with at least one year of lung ultrasound experience) and recorded in patient clinical files together with ultrasound images. LUS scans were performed with micro-linear, hockey stick-shaped, high-frequency (15–18 MHz) probes; machine setting was as previously described.^20^ Given the retrospective design and the routine use of LUS in the recruiting centres, clinicians were not blinded to ultrasound findings. However, LUS aeration score was never used to decide surfactant retreatment, which was only based on FiO_2_ threshold. Moreover, several studies with concealed LUS have demonstrated accurate prediction of the first surfactant dose.^3^^,^^4^ Conversely, due to the nature of the index test, it was impossible to conceal patient conditions, such as vital monitoring and clinical appearance, from physicians performing ultrasound. Nonetheless, previous studies have demonstrated optimal inter-observer agreement with or without blinding of operator.^19^^,^^21^^,^^22^

The reference standard (i.e., the criterion for surfactant retreatment) was the presence of ongoing and persistent oxygen dependency at least 10 h after the first surfactant treatment.^18^ Oxygen dependency was defined as FiO_2_ persistently >0.30 without other causes (e.g., pulmonary hypertension, haemodynamic impairment, anaemia), despite the optimization of respiratory support; the oxygen dependency was considered persistent if it lasted continuously for at least 3 h. This standard is consistent with the criterion for surfactant retreatment suggested by current international guidelines.^14^^,^^23^ To check FiO_2_ needs, pre-ductal SpO_2_ was continuously monitored with artifact filtering systems and considered only when the signal was regularly smooth.

### Statistics

We considered LUS aeration score accurate in predicting the second surfactant treatment, if the area under the curve (AUC) drawn by receiver operator characteristic (ROC) analysis was not inferior to that already known for the prediction of the first surfactant administration.^4^ We choose a target AUC of 0.88 (95% confidence interval (CI): 0.82; 0.91) as this value is given by the summary ROC reported by the latest meta-analysis on the prediction of the first surfactant administration in preterm patients.^4^ We considered AUC = 0.80 as the null hypothesis since this value is associated with an FiO_2_ threshold of 0.30 to indicate the first surfactant dosing,^3^ and the same FiO_2_ threshold was our reference standard.^14^^,^^23^ Thus, the non-inferiority margin was set at 0.08: in other words, we considered the diagnostic accuracy for predicting retreatment to be inferior to that for predicting the first dose if the area under the curve (AUC) was less than 0.80. β- and α-errors were set at 0.9 and at 0.05 (two-sided), respectively. Neonates needing retreatment were considered to be 31% of all preterm neonates, or 43% of those who received a first dose as these prevalences were previously reported by a dedicated European survey on surfactant therapy in a population similar to ours.^24^ The first prevalence value was used to calculate a sample size needed to study diagnostic accuracy of the T1 (i.e., ultrasound performed before any surfactant administration); the second value was used for the T2 score (i.e., ultrasound performed after the first surfactant dose). With these parameters, the sample size needed was 472 (113 who received surfactant retreatment and 359 who received one dose or no surfactant treatment at all) or 390 patients (117 with a repeated and 273 with a single surfactant administration), for T1 and T2 score, respectively.^25^ No interim analysis was planned and the statistical analyses were done at once, after all data collection, as follows.

Clinical and demographic characteristics of the studied population were compared using one-way ANOVA or Kruskal–Wallis test, respectively, followed by *post hoc* Sidak or Conover–Iman test, if needed. ROC analysis was performed to provide AUC and diagnostic accuracy metrics with their 95% CI. The DeLong test^26^ was used to compare AUC between index tests performed at T1 and T2 and those reported for the prediction of the first treatment by the latest meta-analysis of preterm patients^4^ or in the multicentre study recruiting late preterm/term patients.^27^ We anticipated some missing data for the index test: their distribution was studied with the Little test (to verify if the data are “missing completely at random”) and then with logistic regressions accounting for basic clinical characteristics listed in Table 1 to verify if the missingness was linked to any of them. Little test was significant (*p* < 0.001 for both T1 and T2 scores) and regression showed that the missing data were associated with gestational age, CRIB-II and prenatal steroids (all *p* < 0.001, for both T1 and T2 scores). We therefore concluded that data were possibly missing at random,^28^ and ROC analysis was performed after multiple imputation.^29^ We considered AUC as our outcome and evaluated LUS aeration score both as triage and replacement tests. The former consisted in using the cut-off value associated with highest sensitivity, irrespective of specificity; the latter considered the cut-off associated with both highest sensitivity and specificity (i.e., highest global accuracy).^30^ LUS aeration scores of 4 and 8 were the pre-specified positive cut-offs. These values have been respectively associated with the highest sensitivity and highest global accuracy in predicting the first surfactant treatment (i.e., at T1) in neonates of <34 weeks’ gestation^19^ or in those born at ≤ 30 weeks’ gestation.^11^ No data were available about the use of LUS at T2 or anyway as triage test in extremely preterm neonates, therefore we used the data available in the closest population. Both cut-offs are important: ruling out (triage test) would allow to clarify that the oxygen dependency is due to other mechanisms (e.g., haemodynamics, anaemia), while ruling in (replacement test) the retreatment leads to identify neonates with surfactant deficiency causing a reduced lung aeration. These patients should be retreated without further delay because the retreatment has been associated with better oxygenation and clinical outcomes.^10^

**Table 1:** Basic clinical characteristics of the study population.

	Whole population (n = 705)	No surfactant (n = 192)	One surfactant dose (n = 303)	Two surfactant doses (n = 210)	*p*
Gestational age (weeks)	26.6 (1.5)	27.3 (1.3)∗^,†^	26.7 (1.6)∗^,‡^	25.8 (1.5)^†,‡^	<0.001
Birth weight (grams)	900 (257)	1004 (238)∗^,†^	921 (268)∗^,‡^	774 (202)^†,‡^	<0.001
Male sex	346 (49%)	93 (48.4%)	145 (47.9%)	108 (51.4%)	0.760
Prenatal steroids	635 (90%)	184 (95.8%)	268 (88.4%)	183 (87.1%)	<0.001
Clinical chorioamnionitis	197 (27.9%)	61 (31.8%)	74 (24.4%)	62 (29.5%)	0.189
Cord lactate (mmol/L)	3.7 (2.4)	3.2 (1.8)∗	3.6 (2.1)^@^	4.4 (3.2)∗^,@^	<0.001
Caesarean delivery	438 (62.1%)	110 (57.3%)	198 (65.3%)	130 (61.9%)	0.185
5-min Apgar score	8 [6–9]	9 [8–10]^$,O^	7 [6–9]^$,•^	7 [5–8]^O,•^	<0.001
CRIB-II score	9.4 (3.4)	7.8 (3)^#,†^	8.8 (3.4)^#,‡^	11.8 (2.5)^†,‡^	<0.001
T1 LUS aeration score	9 (3.7)	4.6 (2.3)∗^,†^	10 (2.8)∗^,‡^	11.5 (2.4)^†,‡^	<0.001
T2 LUS aeration score	8 (3.6)	3.3 (2.3)∗^,†^	6.9 (3)∗^,‡^	10.5 (2.7)^†,‡^	<0.001

Data are expressed as mean (standard deviation), median [25th – 75th percentile] or number (%), as appropriate. For each column, the numerator is represented by the n on the first row. Prenatal steroids are defined as at least one 12 mg-dose given 24 h before delivery; cord lactate is assayed on arterial blood samples; sex is considered as it was observed at the birth. Ethnicity was not recorded for legal reasons and thus unavailable. Apgar, CRIB-II and LUS aeration score are dimensionless variables. *p*-values refer to the overall one way-ANOVA comparison between neonates receiving zero, one or two surfactant doses. *Post hoc* significant comparisons (Sidak test or Conover–Iman test, as appropriate) are as follows: ∗*p* < 0.001, ^†^*p* < 0.001, ^‡^*p* < 0.001, ^#^*p* = 0.005, ^@^*p* = 0.005, ^$^*p* < 0.05, ^O^*p* < 0.05, ^•^*p* < 0.05.

CRIB-II: critical risk index for babies-II; LUS: lung ultrasound; T1: timepoint 1 (at NICU admission, before surfactant administration if any, i.e., ≈1 h of life); T2: timepoint 2 (at ≈12 h of life and always at least 10 h after the first surfactant administration).

Two procedures were used to verify that diagnostic accuracy was independent of gestational age. First, the aeration scores at T1 and T2 were compared between infants receiving one or two doses using multivariate analysis of covariance (MANCOVA), controlling for gestational age. Second, ROC analysis was repeated for four pre-specified gestational age groups (i.e., 23–24, 25–26, 27–28 and 29–30 weeks’ gestation)^30^ and their AUC values were compared with DeLong test.^26^ These gestational age classes were chosen to have enough cases in each of them considering the typical hospital catchment and recruitment in the participating centres.

Finally, the association between LUS aeration scores and surfactant retreatment was investigated using multivariable binary logistic regression with the enter method.^30^ Covariates were all variables significantly different in the univariate analysis between patients who received one or two doses of surfactant. Results were expressed as adjusted odds ratio (aOR) with 95% CI. Multicollinearity was evaluated as previously described.^31^ Goodness-of-fit was studied with the Hosmer–Lemeshow test, which assesses whether or not the observed event rates match expected event rates in subgroups of the model population (*p*-values > 0.05 indicate a satisfactory model goodness-of-fit). Analyses were performed with SPSS 30 (Armonk-NY, USA) and MedCalc 13.3 (Ostend, Belgium); *p* < 0.05 was considered significant.

### Role of funders

The study was not supported by any funder.

## Results

**Fig. 1:**
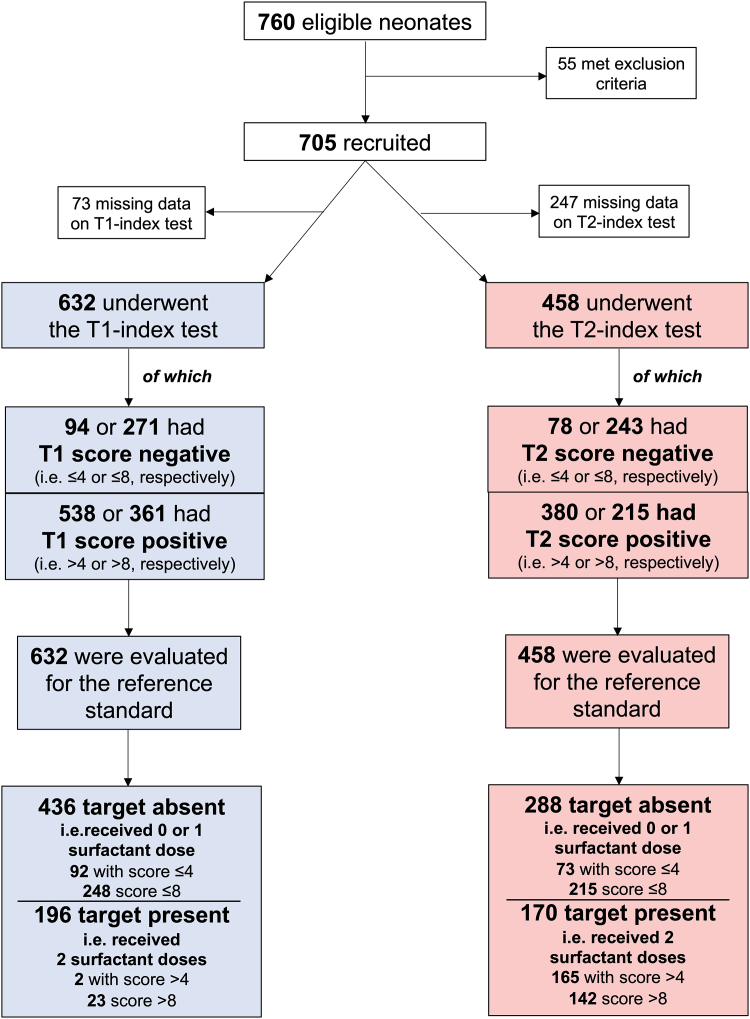
**STARD diagram.** The study flow chart is depicted according to STARD guidelines.^12^ The index test was the LUS aeration score considered at two timepoints, namely T1 and T2, depicted in light blue and pink, respectively. Two pre-specified cut-off values were used for the index test (i.e., > 4 or >8, when using it as triage or replacement, respectively) since these thresholds accurately predicted the first surfactant treatment.^11^^,^^19^ The evaluation for the reference standard was the continued FiO_2_ monitoring. The target was the condition to be predicted, that is, the need for surfactant retreatment. There were no indeterminate results for the index test and the reference standard; there were no missing data for the reference standard (i.e., all enrolled neonates underwent continuous FiO_2_ and clinical monitoring). Missing data for the index test are indicated. The discrepancy between patients with T1-score >8 at upon NICU admission (n = 361) and those actually receiving one surfactant dose (n = 303 in Table 1) is represented by 58 neonates who received surfactant in the delivery room within resuscitation manoeuvres,^14^ before being transferred to the NICU. Criteria used to indicate retreatment, and more details are described in the main text. **Abbreviations**: FiO_2_: inspired oxygen fraction; LUS: lung ultrasound; NICU: neonatal intensive care unit; T1: timepoint 1 (at NICU admission, before surfactant administration if any, i.e., ≈1 h of life); T2: timepoint 2 (at ≈12 h of life and always at least 10 h after the first surfactant administration).

Table 1 describes the study population. Between January 2021 and December 2024, 705 neonates were admitted to the NICU and therefore eligible: given the ease to obtain their data from electronic patient files, they were all recruited to avoid a selection bias. Neonates with no need for surfactant or receiving a single or repeated treatment were obviously different for several characteristics. Median postnatal age at the first surfactant administration was 2 [1–3] and 1 [1–2] h for patients receiving a single or repeated treatment, respectively. Retreatment was provided at a median age of 18 [11–36] h; overall, 572 (81.1%) infants survived and 196 (27.8%) were diagnosed with bronchopulmonary dysplasia. Median NICU stay was 51 [32–72] days. Supplementary Tables S1 and S2 show basic patient data distribution per recruiting centre. Fig. 1 shows the STARD flow chart and describes how many patients had a negative (values ≤ 4 or ≤8, for the triage and replacement test, respectively) or a positive (values > 4 or >8) score at T1 and T2 test. Approximately 10% and 35% of data were missing for the LUS aeration score at T1 and T2, respectively, but there were no indeterminate data for the any of the two LUS aeration scores. There were no missing data for the reference standard. LUS was always performed without any problem or side effect.

**Fig. 2:**
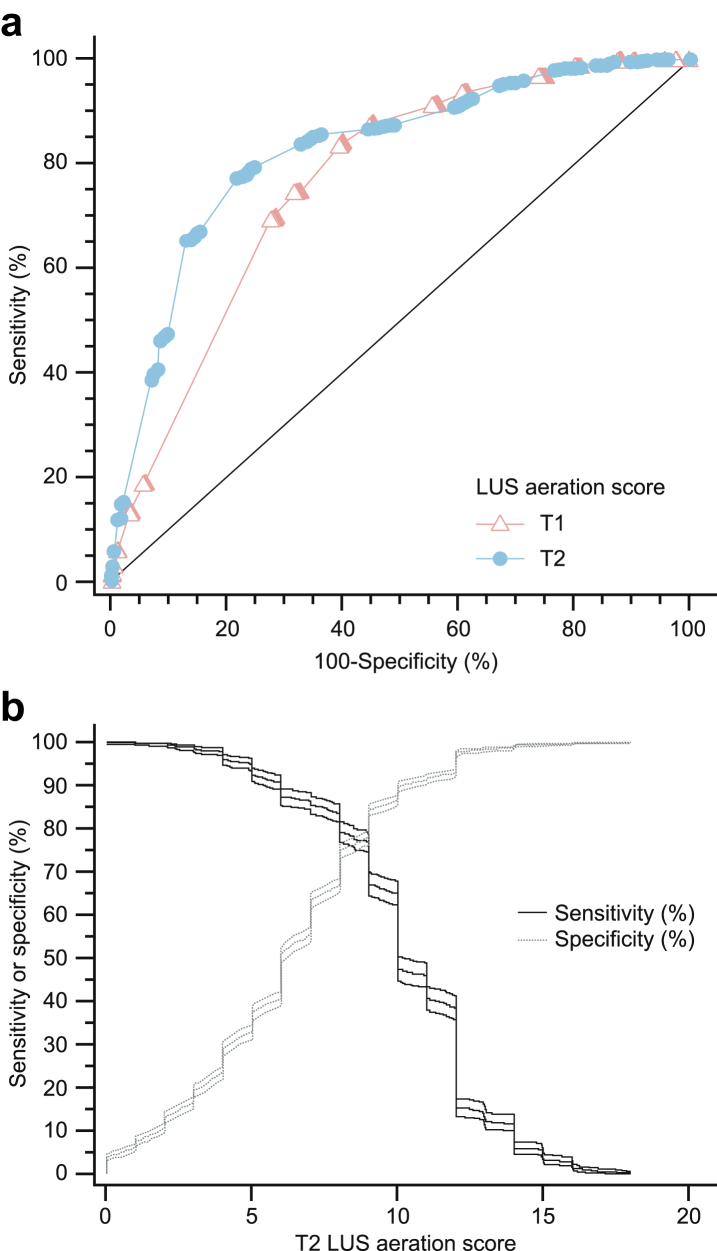
**ROC analysis for surfactant retreatment in preterm neonates who already received a first surfactant dose.** Analysis performed after multiple imputation of missing index test data; n = 2926 after multiple imputation. **Panel A** depicts the comparison between ROC curves for the LUS aeration score calculated at T1 (light blue line, full circles) and at T2 (pink line, open triangles). The area under each curve is significantly different (see text for more details). The black diagonal line represents the reference. **Panel B** visually illustrates the values of sensitivity (thick black line) and specificity (hatched grey line) and their 95% confidence intervals (thin lines) with increasing LUS values: crossing point is approximately at 8. More details in the text. **Abbreviations**: LUS: lung ultrasound; ROC: receiver operator characteristic; T1: timepoint 1 (at NICU admission, before surfactant administration if any, i.e., ≈1 h of life); T2: timepoint 2 (at ≈12 h of life and always at least 10 h after the first surfactant administration).

Fig. 2a shows the ROC curve for T1 and T2 LUS aeration scores: AUC was higher at T2 (AUC = 0.854 (95% CI: 0.84; 0.87), *p* < 0.0001 (DeLong test)) than at T1 (AUC = 0.69 (95% CI:0.67; 0.71), *p* < 0.0001 (DeLong test)); the absolute AUC difference between T2 and T1 (0.165 (95% CI:0.116; 0.214) was statistically significant (*p* < 0.001 (DeLong test)). The AUC of T2 LUS aeration score was not statistically different from that reported for the prediction of the first surfactant treatment by the latest meta-analysis enrolling preterm infants (*p* = 0.225 (DeLong test))^4^ or by the recent multicentre study recruiting late preterm/term patients (*p* = 0.579 (DeLong test)).^27^ Similar results were obtained performing the ROC analysis without multiple imputations and using only complete data (i.e., available case scenario, Supplementary Fig. S1). Fig. 2b confirms that cut-off values associated with the highest sensitivity and highest global accuracy (i.e., both highest sensitivity and specificity) were ≈4 and ≈8, respectively. A cut-off value of 8 gave a Youden index of 0.63. Since the LUS aeration score calculated at T2 performs better, all diagnostic accuracy metrics are reported in Table 2 for the triage and replacement thresholds. The accuracy metrics of all thresholds for both scores are reported in Supplementary Table S3 for comprehensiveness.

**Table 2:** Diagnostic accuracy parameters for the two pre-specified thresholds of T2-LUS aeration score.

Type of test	Cut-off	Sensitivity (95% CI)	Specificity (95% CI)	+LR (95% CI)	−LR (95% CI)	+PV (95% CI)	−PV (95% CI)	Global accuracy (95% CI)	+Post-test probability (95% CI)	−Post-test probability (95% CI)
Triage	4	98% (97; 99)	23% (21; 25)	1.3 (1.2; 1.3)	0.09 (0.06; 0.1)	36% (34; 38)	96% (95; 98)	54% (52; 55)	47% (45; 47)	5% (4; 6)
Replacement	8	84% (82; 86)	70% (69; 73)	2.9 (2.7; 3.1)	0.26 (0.2; 0.3)	56% (54; 58)	90% (89; 91)	78% (74; 79)	67% (65; 68)	15% (12; 17)

These cut-off values were pre-specified to use the LUS aeration score as triage (i.e., with highest sensitivity) or replacement (i.e., highest global diagnostic accuracy, that is both highest sensitivity and specificity). More details in the text. With the exception of LR, all results are rounded to the closest integer.

CI: confidence interval; LR: likelihood ratio; LUS: lung ultrasound score; PV: predictive value.

T1 and T2 LUS aeration scores differed significantly between neonates receiving single or repeated surfactant treatment at the univariate analysis (Table 1). This difference remained statistically significant after controlling for gestational age in MANCOVA (λ = 0.969, *p* = 0.003, η^2^ = 0.31). Subgroup ROC analyses show that for any gestational age class: 1) AUC is higher for the score computed at T2 than at T1 (Supplementary Table S4), and 2) the AUC of the T2 LUS aeration score is similar amongst gestational age classes (Supplementary Table S5).

Finally, with the multivariate analysis, the LUS aeration score calculated at T2 remained the variable more strongly associated with surfactant retreatment (aOR = 1.57 (95% CI:1.38; 1.79), *p* < 0.001 (multivariable logistic regression)). The CRIB-II score was the only other variable significantly associated with surfactant retreatment (OR = 1.44 (95% CI: 1.21; 1.72), *p* < 0.001 (multivariable logistic regression)), while all the other covariates (i.e., LUS at T1, gestational age, 5-min Apgar score, prenatal steroids and arterial cord lactate) were non-significant. No relevant multicollinearity was detected; goodness of fit was satisfactory (*p* = 0.612 (Hosmer–Lemeshow test)).

## Discussion

In a large population of preterm neonates, we found that the diagnostic accuracy of LUS aeration score in predicting surfactant retreatment is: 1) higher if the score is calculated at T2 (i.e., ≈12 h of life and at least 10 h after the first surfactant administration), than at T1 (≈1 h of life, upon NICU admission, that is before any surfactant administration); 2) non-inferior to the diagnostic accuracy known for the first dose prediction, if the score is calculated at T2; and, 3) independent of gestational age, that is the T2 score performs similarly well in any gestational age class and is always superior to the score calculated at T1.

These findings are clinically relevant as they provide guidance to neonatologists using LUS aeration score for the most immature and sick neonates, and answer practical questions about the surfactant retreatment. As the T2 LUS aeration score in identifying patients needing retreatment is non-inferior to that used to predict the first administration, the suggested cut-off values are the same and have similar sensitivity and specificity. Thus, a score >8 in oxygen-dependent preterm neonates with ongoing respiratory failure may be used as replacement test with good accuracy to indicate surfactant retreatment (i.e., the probability of retreating goes from ≈40 to ≈70%) and reduce delayed administration. Conversely, in the same type of patient, a value ≤ 4 used as triage score makes the need for surfactant retreatment unlikely (i.e., the probability of retreating goes from ≈40 to ≈5%). To the best of our knowledge, only two other studies have investigated LUS aeration score to predict surfactant retreatment and showed very similar diagnostic accuracy.^11^^,^^32^ These studies were, however, much smaller, had a single-centre design and one of them^32^ focused on much more mature infants.

We consider our data to be reliable and consistent with the available knowledge. The study strictly followed STARD methodology and was pragmatic as it aimed to capture the clinical reality of surfactant retreatment and investigate the use of quantitative LUS embedded in clinical routine. In fact, in our population, the prevalence of and postnatal age at surfactant retreatment are identical to those described in a dedicated continent-wide survey.^24^ Furthermore, the participating centres shared the same respiratory care policy, provided modern perinatal care, used the same surfactant and had the same LUS protocol and expertise. This identity has helped to provide a homogeneous population and to overcome some of the issues that have hitherto prevented an adequate study of the matter.

The superiority of the score calculated at T2 over the earlier test has a pathobiological plausibility. In fact, the score calculated at T1 (i.e., before surfactant administration, if any) captures the loss of lung aeration present in that phase of respiratory failure and may not accurately predict the later response to the first surfactant administration. Such a response depends on the capability to produce endogenous surfactant and the degree of lung inflammation^9^ which may evolve with time (during which the severity of the disease may change), hence the need to repeat the LUS scan to evaluate the effect. In the future, it would be interesting to study LUS and electrical impedance findings and see which one is more linked to lung inflammation and predict better the need for retreatment.

We must acknowledge some study limitations, mostly related to the retrospective design. First of all, the study was pragmatic as LUS is embedded in our clinical care, therefore the scans were not performed multiple times at fixed timepoints and we cannot know if, for example, a scan 3 or 6 h after the first treatment would be accurate enough. However, our results are easily generalizable, as they were produced within the “real world” practice of three referral centres with the same LUS experience and respiratory protocols. Second, as neonates with acute respiratory distress syndrome (NARDS) show poor response to surfactant^33^ and may have higher LUS aeration scores,^34^^,^^35^ the diagnostic accuracy may be different in these patients; nonetheless, we do not know the proportion of neonates with NARDS in our population and a dedicated study should be performed. Also, we were unable to analyse the effect of mean airway pressure on the need for surfactant redosing: all recruiting centres had the same formal respiratory policy, but, depending on their conditions, patients could have been supported with different techniques. Thus, we cannot exclude that patients with higher pressures would have better lung aeration influencing the diagnostic accuracy. Nevertheless, recent data have demonstrated that, in CPAP-supported neonates, increasing pressure up to 8 cmH_2_O does not significantly change the LUS aeration score.^36^ We used the ongoing respiratory failure with oxygen dependency as reference standard and this cannot be considered a true gold standard: this latter, however, does not exist and our choice is consistent with the criterion recommended by current international guidelines.^14^^,^^23^ The sample size has been calculated assuming that the data were not missing at random, while informative missingness cannot be totally excluded and this assumption may not be directly applied to subgroups with different gestational age. Our findings are however enough strong because the independence from gestational age was verified by several analysis and consistent with the data about the prediction of the first surfactant dose.^11^^,^^19^^,^^27^ We studied a population treated with porcine surfactant which is known to be clinically superior and recommended by European guidelines^14^; results could have been different with bovine surfactants, which are less concentrated and more often require retreatment.^37^ We acknowledge the lack of blinding, given the nature of study intervention (i.e., point-of-care ultrasound), which unavoidably requires observation of the patient and is embedded in our routine clinical care: previous studies have, however, reported good accuracy with various degrees of blinding.^3^^,^^4^^,^^19^^,^^21^^,^^22^ The LUS findings could have unconsciously influenced the clinical decisions, although this is unlikely as the recruiting NICUs have a very strict policy and the respiratory care protocol is enforced with serial refreshes to the whole team. A prospective study with a certain degree of blinding would be helpful to generalise our results. Finally, ethnicity was not recorded, but given our hospital catchments, the studied population can reasonably be considered multiethnic.

In preterm neonates, LUS aeration score calculated at approximately 12 h of life and at least 10 h after the first surfactant administration accurately indicates surfactant retreatment. Its diagnostic accuracy is independent of gestational age and non-inferior to that already reported for the prediction of the first surfactant administration. Cut-off values of 4 and 8 should be considered to use the score as a triage or replacement test, respectively.

## Contributors

Prof. De Luca drafted the manuscript, analysed the data and conceived the project. Drs. Almudena Alonso-Ojembarrena, Davide Sarcina, Irene Gutierrez-Rosa, Barbara Loi, Fiorella Migliaro and Letizia Capasso collected and interpreted the data and critically reviewed the manuscript for important intellectual content; Prof. Francesco Raimondi made substantial contributions to the conception and design of the project and also critically reviewed the manuscript for important intellectual content. Prof. De Luca and Dr. Alonso-Ojembarrena verified the underlying data. All authors approved the final versions to be published, had full access to the data and agreed to be accountable for all aspects of the work in ensuring that questions related to the accuracy or integrity of any part of the work are appropriately investigated and resolved.

## Data sharing statement

The dataset analysed for the study is available, upon the study publication, from the corresponding author on reasonable request respecting all relevant privacy regulations.

## Declaration of interests

Prof. De Luca has served as consultant and lecturer and received research grants or support from Chiesi Farmaceutici and Airway Therapeutics outside the present work. These companies produce surfactant or their components and had no role in this work. Other authors have no conflict of interest to declare.
