# Supplementary material for: Quantitative lung ultrasound to guide surfactant retreatment in preterm neonates born at ≤30 weeks’ gestation: a multicentre retrospective non-inferiority diagnostic accuracy study
Source: eBioMedicine. 2025 Jul 25;118:105865. doi: 10.1016/j.ebiom.2025.105865 (PMC12311550; doi:10.1016/j.ebiom.2025.105865)
Supplement: Supplementary Fig. S1 and Tables S1–S5 [file mmc1.docx]

*Online Supplement*

*for*

**Quantitative lung ultrasound to guide surfactant retreatment in preterm neonates born at ≤30 weeks’ gestation: a multicentre retrospective non-inferiority diagnostic accuracy study**

*Prof. Daniele De Luca (PhD),^1,2^ Almudena Alonso-Ojembarrena (PhD),^3,4^*

*Davide Sarcina (MD),^1^ Irene Gutierrez-Rosa (MD),^3,4^ Barbara Loi (MD),^1,2^*

*Fiorella Migliaro (MD),^5^ Letizia Capasso (MD)^5^ and Prof. Francesco Raimondi (PhD)^5^*

*1 Division of Paediatrics and Neonatal Critical Care, “A. Béclère” Hospital, APHP-Paris Saclay University (Paris, France)*

*2 Physiopathology and Therapeutic Innovation Unit-INSERM U999,*

*Paris Saclay University (Paris, France)*

*3 Neonatal Intensive Care Unit, Puerta del Mar University Hospital (Cádiz, Spain) 4 Biomedical Research and Innovation Institute of Cádiz (INiBICA) Research Unit, Puerta del Mar University Hospital (Cádiz, Spain)*

*5 Division of Neonatology, Department of Translational Medical Sciences, University of Naples Federico II (Naples, Italy)*

**eTable 1. Main basic patient data distribution per recruiting center.** Data are expressed as mean (standard deviation), median [25^th^ – 75^th^ percentile] or number (%), as appropriate. Prenatal steroids are defined as at least one 12-mg dose given 24 h before delivery; cord lactate is assayed on arterial blood samples; Apgar score is a dimensionless variable. Recruiting centers are anonymized.

|  | **A (n=482)** | **B (n=102)** | **C (n=121)** |
| --- | --- | --- | --- |
| Gestational age (weeks) | 26.3 (1.4) | 27.7 (1.6) | 26.9 (1.8) |
| Birth weight (grams) | 861 (222) | 1008 (299) | 970 (297) |
| Male sex | 229 (47.5%) | 52 (50.9%) | 65 (53.7%) |
| Prenatal steroids | 436 (90.5%) | 93 (91.2%) | 106 (79.9%) |
| Clinical chorioamnionitis | 168 (34.8%) | 9 (8.8%) | 20 (16.5%) |
| Cord lactate (mmol/L) | 3.6 (2.4) | 1.6 (0.1) | 4 (2.5) |
| Cesarean delivery | 283 (58.7%) | 83 (81.4%) | 72 (59.5%) |
| 5-minute Apgar score | 8 [6-9] | 8 [7-8] | 7 [6-8] |

**eTable 2. Sex-specific distribution of the main basic patient data.** Data are expressed as mean (standard deviation), median [25^th^ – 75^th^ percentile] or number (%), as appropriate. For each case reporting a dichotomic variable, the numerator is represented by the sex-specific n shown in the first or second row. Prenatal steroids are defined as at least one 12 mg-dose given 24 h before delivery; cord lactate is assayed on arterial blood samples; sex is considered as it was observed at the birth. Ethnicity was not recorded for legal reasons and thus unavailable. Apgar, CRIB-II and LUS aeration score are dimensionless variables.

|  | **Whole**  **population** | **No**  **surfactant** | **One**  **surfactant dose** | **Two**  **surfactant doses** |
| --- | --- | --- | --- | --- |
| **Males** | **346** | **93** | **145** | **108** |
| **Females** | **359** | **99** | **158** | **102** |
|  | | | | |
| **Gestational age (weeks)** | | | | |
| Males | 26.6 (1.6) | 27.3 (1.2) | 26.8 (1.6) | 25.8 (1.6) |
| Females | 26.6 (1.6) | 27.3 (1.3) | 26.7 (1.5) | 25.7 (1.4) |
| **Birth weight (grams)** | | | | |
| Males | 933 (268) | 1041 (255) | 962 (273) | 799 (214) |
| Females | 872 (268) | 970 (216) | 891 (253) | 749 (186) |
| **Prenatal steroids** | | | | |
| Males | 310 (89.6%) | 90 (96.7%) | 130 (89.6%) | 91 (84.2%) |
| Females | 324 (90.2%) | 94 (94.9%) | 138 (87.3%) | 92 (90.2%) |
| **Clinical chorioamnionitis** | | | | |
| Males | 92 (26.6%) | 34 (36.6%) | 29 (20%) | 33 (30.6%) |
| Females | 107 (29.8%) | 27 (27.3%) | 44 (27.8%) | 29 (28.4%) |
| **Cord lactate (mmol/L)** | | | | |
| Males | 3.8 (2.7) | 3.1 (1.6) | 3.6 (2.4) | 4.6 (3.5) |
| Females | 3.6 (2.2) | 3.3 (1.8) | 3.5 (1.9) | 4.2 (2.7) |
| **Caesarean delivery** | | | | |
| Males | 217 (62.7%) | 56 (60.2%) | 96 (66.2%) | 65 (60.2%) |
| Females | 223 (62.1%) | 54 (54.5%) | 101 (63.9%) | 65 (63.7%) |
| **5-minute Apgar score** | | | | |
| Males | 8 [6-9] | 8 [8-9] | 8 [6-9] | 7 [5-8] |
| Females | 7 [6-9] | 9 [8-10] | 7 [5-9] | 6 [3-8] |
| **CRIB-II score** | | | | |
| Males | 9.4 (3.6) | 7.6 (3.2) | 8.8 (3.6) | 11.9 (2.5) |
| Females | 9.3 (3.3) | 8 (2.8) | 8.8 (3.2) | 11.6 (2.5) |
| **T1 LUS aeration score** | | | | |
| Males | 8.9 (3.8) | 4.4 (2.5) | 10 (2.5) | 11.3 (2.3) |
| Females | 9 (3.8) | 4.9 (2.2) | 10 (3) | 11.6 (2.4) |
| **T2 LUS aeration score** | | | | |
| Males | 8.1 (3.8) | 3.7 (2.5) | 6.8 (3.2) | 10.8 (2.8) |
| Females | 8 (3.4) | 2.9 (2) | 7 (2.9) | 10.3 (2.6) |

**eFigure 1. ROC analysis for surfactant retreatment in extremely preterm neonates who already received a first surfactant dose.** Analysis performed as per available case scenario (n=366). The figure shows the comparison between ROC curves for the LUS aeration score calculated at T1 (light blue line, full circles) and at T2 (pink line, open triangles). The black diagonal line represents the reference. The AUC is significantly different (*p*<0.0001 (DeLong test)) between T1 (AUC = 0.656 (95%CI: 0.603;0.710), *p*<0.001 (DeLong test)) and T2 (AUC = 0.839 (95%CI: 0.797; 0.882), *p*<0.001 (DeLong test)). **Abbreviations**: AUC: area under the curve; LUS: lung ultrasound; ROC: receiver operator characteristic; T1: timepoint 1 (at NICU admission, before surfactant administration if any, i.e. ≈1h of life); T2: timepoint 2 (at ≈12h of life and always at least 10h after the first surfactant administration).

**
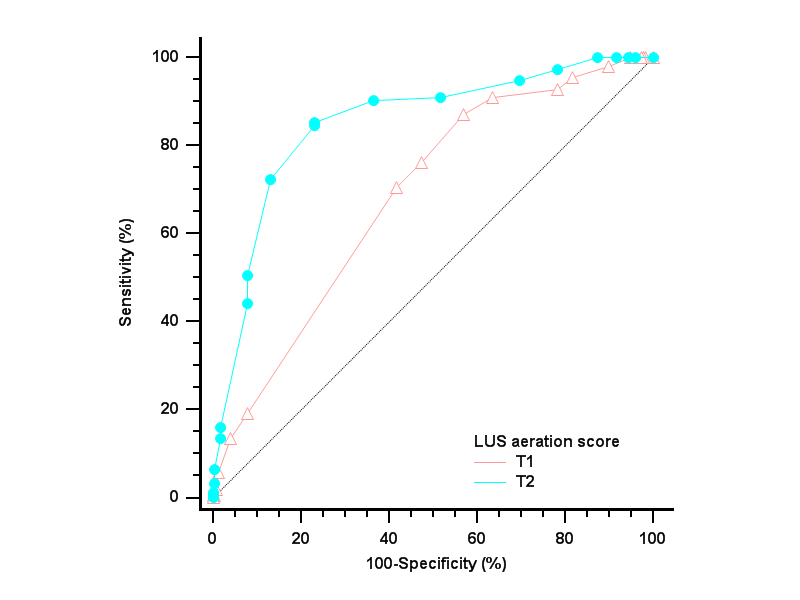
**

**eTable 3. Main diagnostic accuracy parameters of all thresholds for LUS aeration score calculated at T1 and T2. Abbreviations**: LR: likelihood ratio; LUS: lung ultrasound; n.c.: non calculated; Prob: probability; PV: predictive value; Sens: sensitivity; Spec: specificity; T1: timepoint 1 (at NICU admission, before surfactant administration if any, i.e. ≈1 h of life); T2: timepoint 2 (at ≈12 h of life and always at least 10 h after the first surfactant administration).

|  | **LUS aeration score at T1** | | | | | | | | | **LUS aeration score at T2** | | | | | | | | |
| --- | --- | --- | --- | --- | --- | --- | --- | --- | --- | --- | --- | --- | --- | --- | --- | --- | --- | --- |
| **Cut-off value** | **Sens**  **(%)** | **Spec**  **(%)** | **Youden**  **index** | **+LR** | **+LR** | **+PV**  **(%)** | **-PV**  **(%)** | **+Post test**  **Prob** | **-Post test**  **Prob** | **Sens**  **(%)** | **Spec**  **(%)** | **Youdenindex** | **+LR** | **+LR** | **+PV**  **(%)** | **-PV**  **(%)** | **+Post test**  **Prob** | **-Post test**  **Prob** |
| **1** | 100 | 2.5 | 0.04 | 1.03 | 0 | 30.3 | 100 | 30.4 | n.c | 99.9 | 8 | 0.08 | 1.09 | 0.01 | 31.6 | 99.5 | 31.6 | 0.4 |
| **2** | 100 | 7.6 | 0.08 | 1.08 | 0 | 31.5 | 100 | 31.4 | n.c | 99.7 | 13 | 0.13 | 1.15 | 0.02 | 32.7 | 99 | 32.8 | 0.8 |
| **3** | 100 | 12 | 0.12 | 1.14 | 0 | 32.5 | 100 | 32.6 | n.c | 98.5 | 19 | 0.18 | 1.22 | 0.08 | 34 | 96.8 | 34.1 | 3.3 |
| **4** | 99.5 | 17 | 0.17 | 1.2 | 0.03 | 33.7 | 98.8 | 33.7 | 1.2 | 98 | 23 | 0.25 | 1.27 | 0.09 | 35.1 | 96.4 | 35 | 3.7 |
| **5** | 98 | 24 | 0.22 | 1.29 | 0.08 | 35.4 | 96.6 | 35.8 | 3.2 | 92.5 | 37.6 | 0.3 | 1.48 | 0.2 | 38.6 | 92.2 | 38.6 | 7.8 |
| **6** | 95.4 | 34 | 0.29 | 1.45 | 0.14 | 38 | 94.6 | 38.1 | 5.6 | 87.5 | 50.9 | 0.38 | 1.78 | 0.25 | 43.1 | 90.6 | 43 | 9.6 |
| **7** | 93 | 44 | 0.37 | 1.66 | 0.16 | 41.3 | 93.7 | 41.3 | 6.4 | 85.6 | 63.5 | 0.49 | 2.35 | 0.23 | 49.9 | 91.2 | 49.9 | 8.9 |
| **8** | 90 | 52 | 0.42 | 1.88 | 0.19 | 44.3 | 92.5 | 44.4 | 7.5 | 84 | 70 | 0.63 | 2.8 | 0.23 | 54.3 | 91.2 | 54.3 | 8.9 |
| **9** | 86.7 | 59.3 | 0.46 | 2.13 | 0.22 | 47.5 | 91.3 | 47.5 | 8.5 | 67.1 | 84.3 | 0.51 | 4.27 | 0.39 | 64.5 | 85.8 | 64.5 | 14.2 |
| **10** | 80.8 | 65.3 | 0.46 | 2.33 | 0.29 | 49.7 | 88.9 | 49.7 | 10.9 | 47.5 | 90 | 0.37 | 4.75 | 0.58 | 66.9 | 80.1 | 66.9 | 19.8 |
| **11** | 74.2 | 70.5 | 0.45 | 2.52 | 0.37 | 51.6 | 86.6 | 51.7 | 13.6 | 40.7 | 91.7 | 0.32 | 4.9 | 0.65 | 67.5 | 78.5 | 67.5 | 21.6 |
| **12** | 45.2 | 84 | 0.29 | 2.83 | 0.65 | 54.33 | 78.3 | 54.6 | 21.6 | 15.3 | 97.8 | 0.13 | 6.95 | 0.87 | 74.7 | 73.1 | 74.7 | 27 |
| **13** | 15.6 | 96.2 | 0.12 | 4.11 | 0.88 | 63.54 | 72.9 | 63.6 | 27.2 | 12.1 | 98.3 | 0.10 | 7.12 | 0.89 | 75.1 | 72.5 | 75.1 | 27.4 |
| **14** | 9.7 | 98.3 | 0.08 | 5.71 | 0.92 | 70.8 | 71.9 | 70.8 | 28.1 | 5.9 | 99.5 | 0.05 | 11.8 | 0.95 | 83.4 | 71.3 | 83.4 | 28.7 |
| **15** | 4.6 | 99.4 | 0.04 | 7.67 | 0.96 | 76.5 | 71 | 76.5 | 28.9 | 3.1 | 99.6 | 0.03 | 7.75 | 0.97 | 76.7 | 70.8 | 76.7 | 29.2 |
| **16** | 2.3 | 99.7 | 0.02 | 7.67 | 0.98 | 76.5 | 70.6 | 76.5 | 29.4 | 1.3 | 100 | 0.01 | n.c. | 0.99 | 100 | 70.5 | n.c. | 29.6 |
| **17** | 1.5 | 99.9 | 0.01 | 15 | 0.99 | 86.4 | 70.5 | 86.4 | 29.6 | 0.6 | 100 | 0.009 | n.c. | 0.99 | 100 | 70.3 | n.c. | 29.6 |
| **18** | 0.7 | 100 | 0.008 | n.c | 0.99 | 100 | 70.3 | n.c. | 29.6 | 0.3 | 100 | 0.003 | n.c. | 0.99 | 100 | 70.2 | n.c. | 29.6 |

**eTable 4. Subgroup ROC analyses comparing LUS aeration scores calculated at T1 and T2 according to pre-specified gestational age classes.** *p*-values refer to the paired T1-T2 comparisons realized with the DeLong test (n=2926 after multiple imputation). Results for neonates of 29- and 30-weeks’ gestation are not shown because only one positive case was present in this class (i.e. only one patient of this age needed surfactant retreatment). Gestational age classes were chosen to have enough cases in each of them considering the typical hospital catchment and recruitment in the participating centers. **Abbreviations**: ΔAUC: T2 – T1 difference in AUC; AUC: area under the curve; ROC: receiver operator characteristic; T1: timepoint 1 (at NICU admission, before surfactant administration if any, i.e. ≈1h of life); T2: timepoint 2 (at ≈12 h of life and always at least 10 h after the first surfactant administration).

|  | **AUC (95%CI)** | | Δ**AUC** | ***p*** |
| --- | --- | --- | --- | --- |
|  | **T1** | **T2** |  |  |
| **23-24**  **weeks** | 0.692  (0.549-0.835) | 0.742  (0.600-0.885) | 0.05 | 400 |
| **25-26**  **weeks** | 0.728  (0.647-0.808) | 0.848  (0.783-0.912) | 120 | 3 |
| **27-28**  **weeks** | 0.709  (0.632-0.787) | 0.910  (0.859-0.961) | 201 | <0.001 |

**eTable 5. Subgroup ROC analyses comparing the AUC of T2-LUS aeration scores in pre-specified gestational age classes.** *p*-values refer to the comparison of the AUC between rows and columns (i.e. between different gestational age classes), realized with the DeLong test (n=2926 after multiple imputation). For each comparison, the ΔAUC (with 95%CI) and *p*-value are shown. Gestational age classes were chosen to have enough cases in each of them considering the typical hospital catchment and recruitment in the participating centers. **Abbreviations**: ΔAUC: difference in AUC between two gestational age classes; AUC: area under the curve; ROC: receiver operator characteristic; T2: timepoint 2 (at ≈12h of life and always at least 10 h after the first surfactant administration).

|  | Δ**AUC (95%CI)** | | | |
| --- | --- | --- | --- | --- |
|  | **23-24**  **weeks** | **25-26**  **weeks** | **27-28**  **weeks** | **29-30 weeks** |
| **23-24**  **weeks** |  | -0.105  (-0.261; 0.051)  *p*=0.186 | -0.168  (-0.319; -0.017)  *p*=0.05 | -0.124  (-0.347; 0.099)  *p*=0.275 |
| **25-26**  **weeks** | -0.105  (-0.261; 0.051)  *p*=0.186 |  | -0.058  (-0.146; 0.03)  *p*=0.199 | -0.085  (-0.201; 0.032)  *p*=0.157 |
| **27-28**  **weeks** | -0.168  (-0.319; -0.017)  *p*=0.05 | 0.058  (-0.146; 0.03)  *p*=0.199 |  | -0.027  (-0.142; 0.089)  *p*=0.649 |
| **29-30**  **weeks** | -0.124  (-0.347; 0.099)  *p*=0.275 | -0.085  (-0.201; 0.032)  *p*=0.157 | -0.027  (-0.142; 0.089)  *p*=0.649 |  |
